# Imaging-based body fat depots and new-onset atrial fibrillation in general population: a prospective cohort study

**DOI:** 10.1186/s12916-022-02505-y

**Published:** 2022-09-19

**Authors:** Zuolin Lu, Martijn J. Tilly, Elif Aribas, Daniel Bos, Sven Geurts, Bruno H. Stricker, Robert de Knegt, M. Arfan Ikram, Natasja M. S. de Groot, Trudy Voortman, Maryam Kavousi

**Affiliations:** 1 Department of Epidemiology, Erasmus MC, office Na-2714, University Medical Centre Rotterdam, PO Box 2040, 3000 CA Rotterdam, The Netherlands; 2 Department of Radiology and Nuclear Medicine, Erasmus MC, Erasmus University Medical Centre Rotterdam, Rotterdam, The Netherlands; 3 Department of Gastroenterology & Hepatology, Erasmus MC, Erasmus University Medical Centre Rotterdam, Rotterdam, The Netherlands; 4 Department of Cardiology, Erasmus MC, Erasmus University Medical Centre Rotterdam, Rotterdam, The Netherlands

**Keywords:** Atrial fibrillation, Fat depots, Dual-energy X-ray absorptiometry, Computed tomography

## Abstract

**Background:**

Obesity is a well-established risk factor for atrial fibrillation (AF). Whether body fat depots differentially associate with AF development remains unknown.

**Methods:**

In the prospective population-based Rotterdam Study, body composition was assessed using dual-energy X-ray absorptiometry (DXA) and liver and epicardial fat using computed tomography (CT). A body composition score was constructed by adding tertile scores of each fat depot. Principal component analysis was conducted to identify potential body fat distribution patterns. Cox proportional hazards regression was used to calculate hazard ratios and 95% confidence intervals (HR; 95% CI) per 1-standard deviation increase in corresponding fat depots to enable comparisons.

**Results:**

Over a median follow-up of 9.6 and 8.6 years, 395 (11.4%) and 172 (8.0%) AF cases were ascertained in the DXA and the CT analyses, respectively. After adjustments for cardiovascular risk factors, absolute fat mass (HR; 95% CI 1.33; 1.05–1.68), gynoid fat mass (HR; 95% CI 1.36; 1.12–1.65), epicardial fat mass (HR; 95% CI 1.27; 1.09-1.48), and android-to-gynoid fat ratio (HR; 95% CI 0.81; 0.70-0.94) were independently associated with new-onset AF. After further adjustment for lean mass, associations between fat mass (HR; 95% CI 1.17; 1.04-1.32), gynoid fat mass (HR; 95% CI 1.21; 1.08–1.37), and android-to-gynoid fat ratio (HR; 95% CI 0.84; 0.72–0.97) remained statistically significant. Larger body fat score was associated with a higher AF risk (HR; 95% CI 1.10; 1.02–1.20). Borderline significant association was found between a subcutaneous fat predominant pattern with AF onset (HR; 95% CI 1.21; 0.98–1.49).

**Conclusions:**

Various body fat depots were associated with new-onset AF. Total fat mass and gynoid fat mass were independently associated with AF after adjustment for body size. The inverse association between android-to-gynoid fat ratio with AF presents a novel finding. A significant dose-response relationship between body fat accumulation and AF was observed. Our results underscore the predominant role of subcutaneous fat on AF development among a middle-aged and elderly population.

**Graphical abstract:**

Associations betw2een body fat depots, fat distribution and new-onset atrial fibrillation. Abbreviations: AF, atrial fibrillation.
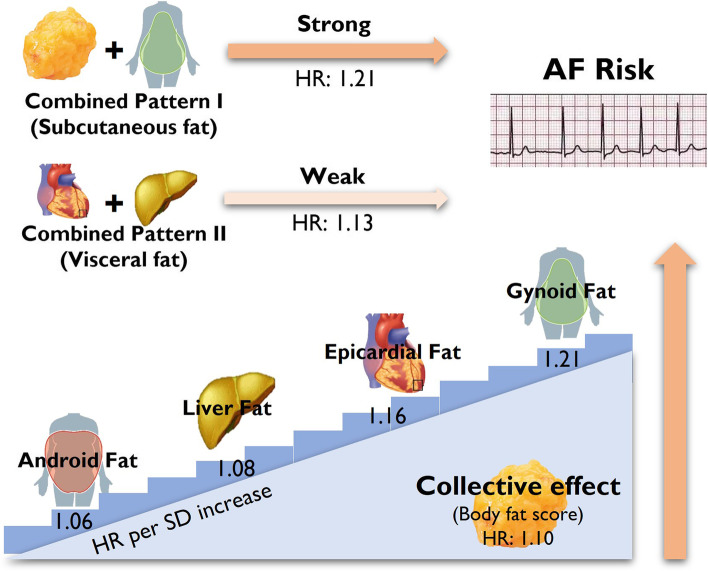

**Supplementary Information:**

The online version contains supplementary material available at 10.1186/s12916-022-02505-y.

## Background

Atrial fibrillation (AF) is the most common cardiac arrhythmia and the currently estimated worldwide prevalence of AF in adults is above 2% [1, 2]. At 55 years of age, the remaining lifetime risk of AF is estimated at 37% among individuals of European ancestry [3].

Obesity is a well-established risk factor for AF [3–7]. Beyond the widely recognized association of higher body mass index (BMI) with increased risk of AF, associations of lean body mass, but weak or no independent associations of fat mass, with higher risk of AF have been reported [8–11]. This has called for further investigation regarding the role of body size in AF studies. However, a recent Mendelian randomization study reported a causal relationship between both fat mass and lean mass with incident AF [12]. Moreover, emerging evidence has indicated links between body fat depots, such as liver fat and epicardial fat, with incident AF, but the results remain inconsistent [13–15]. This could imply a potential role for various body fat depots, beyond total fat mass, on AF development. As obesity may contribute to increased AF risk via inflammatory response of excess adipose tissue [16, 17], different fat depots may exert differential impact on cardiac pathology.

AF and aging are closely intertwined and the prevalence and incidence of AF increase sharply after 65 years of age [18]. The larger risk of AF among the elderly could partly be accounted for by presence of comorbidities and variations in the metabolic profile [19]. Meanwhile, decline in levels of sex hormones critically influences the body fat distribution and physiological function of adipose tissues with aging [20]. However, few studies have comprehensively assessed the impact of various body fat depots and fat distribution on AF among the elderly. Given that advancing age is the most significant and unmodifiable risk factor for AF onset, evaluation of risk factors and devising screening strategies for AF, targeting the elderly, is of clinical importance.

Taking advantage of availability of several body fat measures assessed through dual-energy X-ray absorptiometry (DXA) and computed tomography (CT) within the population-based Rotterdam Study, we aimed to prospectively investigate the associations between various body fat depots and risk of new onset. Given a potential impact of body size in AF onset, we aimed to explore the independent role of total fat mass and various fat depots on AF by adjusting for total lean mass. We further examined whether individual fat depots provide additional information on AF risk beyond total fat mass. Also, the impact of potential body fat distribution patterns on incident AF was investigated.

## Methods

### Study population

The present study was embedded in the Rotterdam Study (RS) [21]. The Rotterdam Study is a prospective population-based cohort study that aims to assess the occurrence and progression of risk factors for chronic diseases in middle-age and elderly persons. During 1990–1993, all inhabitants of Ommoord, a district in the city of Rotterdam, the Netherlands, aged ≥ 55 years were invited for the study. A total of 7983 (78% of all invitees) agreed to participate (RS-I). In 2000, the cohort was extended with 3011 new participants who had become ≥ 55 years or had migrated into the research area (RS-II). Participants attend followed-up examinations every 3–4 years. Outcome data on morbidity and mortality were continuously collected through linkage with digital files from general practitioners in the study area [21].

The Rotterdam Study has been approved by the Medical Ethics Committee of Erasmus MC (registration number MEC 02.1015) and by the Dutch Ministry of Health, Welfare, and Sport (Population Screening Act WBO, license number 1071272-159521-PG). The Rotterdam Study has been entered into the Netherlands National Trial Register (NTR; www.trialregister.nl) and into the WHO International Clinical Trials Registry Platform (ICTRP; www.who.int/ictrp/network/primary/en/) under shared catalog number NTR6831. All participants provided written informed consent to participate in the study and to have their information obtained information from their treating physicians.

**Fig. 1:**
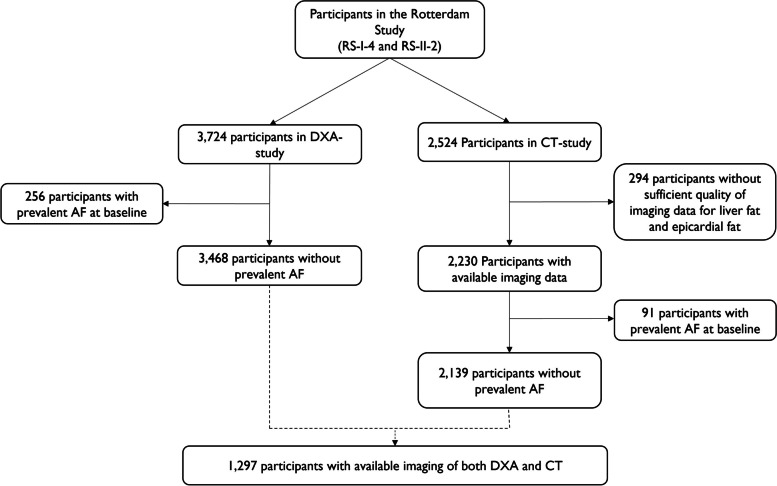
Flowchart of the study population

Body composition was assessed through DXA during the fourth visit of RS-I (RS-I-4) and the second visit of RS-II (RS-II-2), between 2002 and 2006 [22]. In total, 3724 participants were scanned by DXA. We excluded participants with prevalent AF at baseline (*N* = 256). Thus, 3468 individuals were included in the analyses for DXA-assessed fat depots and incident AF. From 2003 to 2006, all participants who visited the research center were asked to participate in a computed tomography (CT) study aimed at visualizing vascular calcification in multiple vessel beds. In total, 2524 participants were scanned by CT [23]. Among 2230 participants with sufficient quality CT scan (*N*-excluded = 294), 91 participants with prevalent AF at baseline were excluded. In total, 2139 participants were included in the CT-assessed fat depot analyses. Further analyses of body composition score and fat distribution in association with AF risk included participants who had both DXA and CT measurements available (*n* = 1297). (Fig. 1)

### Assessment of body composition

Body composition was assessed by DXA using a Prodigy^TM^ total body-fan beam densitometer (GE Lunar Corp, Madison, WI, USA) following the manufacturer protocols, with scans analyzed with enCORE software V13.6 (from GE Lunar) using pre-determined regions of interest. Total lean mass is the sum of trunk lean mass and appendicular lean mass (the sum of lean tissue from the arms and legs), and total fat mass is the sum of android fat mass (localized around the waist), gynoid fat mass (localized around the breasts, hips, and thighs), and fat mass not otherwise specified [22, 24]. As reported, repeatability of the DXA-based measurements was excellent with a Pearson correlation coefficient of 0.991 for total fat mass and 0.994 for total lean mass [25]. The android-to-gynoid fat mass ratio was also calculated. In addition to the net mass of each fat depot, we calculated the percentage of fat mass, android fat mass, and gynoid fat mass as well by means of dividing each of them by total body mass.

### Assessment of liver fat and epicardial fat

A 16-slice (*n* = 785) or 64-slice (*n* = 1739) multidetector CT scanner (Somatom Sensation 16 or 64, Siemens, Forchheim, Germany) was used to perform non-enhanced cardiac, ECG-gated CT scanning. Detailed information regarding imaging parameters of the scans is provided elsewhere [23].

Liver fat was assessed by using a standardized procedure which was described previously [23]. In brief, we placed three circular regions of interest in the liver using Philips iSite Enterprise software (Royal Philips Electronics N.V. 2006) [26]. These regions were drawn throughout the imaged liver tissue (including both the left and right liver lobes) and were carefully chosen to include only liver tissue, and no disruptive tissue such as large blood vessels, cysts, or focal lesions. Then, we calculated the mean Hounsfield unit (HU) value from these three measurements as a marker of the total amount of liver fat, which is a reliable proxy for the mean HU value of the whole liver [26]. We used a fully automatic method [27] to quantify the amount of epicardial fat in milliliters. In short, this method consisted of whole heart segmentation and epicardial fat volume calculation. For the segmentation, we used a multi-atlas-based approach, in which eight manually segmented contrast-enhanced cardiac scans (atlases) were registered (spatially aligned) with every participant’s CT scan. Next, we used this segmentation to determine the amount of epicardial fat.

### Assessment of atrial fibrillation

Methods on event adjudication for prevalent and incident AF have been described previously [13, 22, 28, 29].. Ascertainment of AF at baseline and follow-up examinations in our study has been based on clinical information from the medical records for all participants of the Rotterdam Study. Within the Rotterdam Study, data on medical history and medication use are continuously being collected through multiple sources including a baseline home interview, a physical examination at our research center, the pharmacy prescription records, the Nationwide Medical Registry of all primary and secondary hospital discharge diagnosis, and screening of general practitioner’s records. In addition, a resting 10-s 12-lead electrocardiogram (ECG) used with an ACTA Gnosis IV ECG recorder (Esaote; Biomedical, Florence Italy) is obtained from all participants at every visit of the Rotterdam Study to verify AF. The ECG records were stored digitally and analyzed with the Modular ECG Analysis system (MEANS). The ECG records were stored digitally and analyzed with the Modular ECG Analysis system (MEANS). Subsequently, the AF outcomes are adjudicated independently by two research physicians. In case of disagreement, a senior cardiologist is consulted. Participants were followed from the date of enrolment in the RS until the date of onset of AF, date of death, loss to follow-up, or to January 1, 2014, whichever occurred first.

### Assessment of cardiovascular risk factors

Methods for assessment of cardiovascular risk factors are detailed in Additional file 1: Methods [22, 28, 30].

### Statistical analysis

Descriptives were presented as mean ± standard deviation (SD) or median (interquartile range—IQR) for continuous variables and numbers (percentages) for categorical variables. As the HU values (*A*) had a left-skewed, non-normal distribution, we used exponential transformed values (*B*) [*B* = *A*^3.5^/10,000] [23]. Also, since a lower value of HU represents a larger amount of liver fat, we inversed the HU values (-HU) to represent levels of liver fat.

Values of each fat depot were standardized to allow for direct comparisons. Cox proportional hazards regression analysis was used to examine whether various measures of fat depot including fat mass, android fat mass, gynoid fat mass, android to gynoid fat ratio, liver fat, and epicardial fat were associated with the new-onset AF. Besides, in the analysis with DXA measures, we also assessed each percentage of fat mass, android fat mass, and gynoid fat mass of total body mass in association with incident AF. In the first model, we calculated the age- and sex-adjusted hazard ratio (HR) and their 95% confidence intervals (CIs). Model 2 was additionally adjusted for cardiovascular risk factors including total and HDL cholesterol, history of hypertension, history of diabetes mellitus (DM), history of coronary heart disease (CHD), history of heart failure (HF), history of left ventricular hypertrophy, smoking status, total alcohol intake, use of lipid-lowering medication, and use of cardiac medication. Moreover, we additionally adjusted for total lean mass in Model 3 and for total fat mass in Model 4, respectively. Finally, potential nonlinear associations were examined between various fat depots and incident AF using splines analyses in the Cox models. However, there was no indication for significant nonlinearity (all *P* for nonlinearity >0.05, result not shown). In sensitivity analyses, effect modification by sex was tested. Besides, the analyses were repeated after stratifying participants by BMI; categories of BMI<25 and BMI≥25 kg/m^2^. We also preformed all analyses among participants without prevalent cardiovascular diseases (CHD, HF, and stroke) at baseline.

To investigate the potential cumulative effect of fat depots, we further included the five fat depots (fat mass, android fat mass, gynoid fat mass, liver fat, and epicardial fat) to generate a body fat score. Each fat depot was scored from 0 to 2 according to the respective tertile. All component scores were summed to obtain a total score ranging from 0 to 10 in our population, with higher scores indicating higher total body fat. Consequently, Cox proportional hazards regression analyses, with the same multivariate-adjusted models as aforementioned, were conducted to investigate the impact of body fat score (as tertiles: score 0–2, score 3–6, and score 7–10 with the first tertile as the reference group) on incident AF.

We then developed fat distribution patterns. The distribution patterns were derived using principal component analysis (PCA) on values of the fat depots (including fat mass, android fat mass, gynoid fat mass, liver fat, and epicardial fat) [31]. We used varimax rotation to obtain potential principal components. Factor loadings, which reflect the standardized correlation between each fat depot and a body fat distribution pattern, were used to characterize potential patterns using a cut-off of 0.5. For each participant, pattern-adherence scores were constructed by summing up observed values of the pattern’s fat depots weighted by the corresponding factor loading for each of the two patterns separately. Further, Cox proportional hazards regression analysis was used to assess how the identified fat distribution patterns (as quartiles with the first quartile as reference) were associated with incident AF.

All missing values in covariates were imputed under the assumption of missing at random using multiple imputation [32]. For multiple imputation, all available data was used to generate 5 imputed data sets. Statistical significance was considered at two-tailed *P*-value <0.05. The analyses were done using R software (R 4.0.3; R Foundation for Statistical Computing, Vienna, Austria).

## Results

The baseline characteristics are shown in Table 1. The mean age (SD) of participants in DXA study and CT study were 74.4 (6.8) and 68.7 (6.4) years, respectively. During follow-up, 395 (11.4%) participants in the DXA analysis and 172 (8.0%) participants in the CT analysis developed incident AF. The AF incidence rate was 13.1 per 1000 person-years and 9.9 per 1000 person-years in the DXA and in the CT analyses, respectively.

**Table 1:** Baseline characteristics of the participants

	Imaging method	
	DXA analysis	CT analysis
Number of participants	3468	2139
Follow-up time (years)	9.6 (7.6–10.6)	8.6 (7.9–9.2)
Age (years)	72.4 (6.8)	68.7 (6.4)
Sex (female), *N* (%)	2 038 (58.8)	1150 (53.8)
Body mass index (kg/m^2^)	27.5 (4.0)	27.8 (4.0)
Systolic blood pressure (mmHg)	150.6 (21.1)	146.8 (20.1)
Diastolic blood pressure (mmHg)	80.1 (10.8)	80.3 (10.8)
Total cholesterol (mmol/L)	5.65 (0.98)	5.69 (0.98)
HDL cholesterol (mmol/L)	1.45 (0.39)	1.44 (0.39)
Waist-to-hip ratio	0.90 (0.09)	0.91 (0.09)
Alcohol intake (g/day)	11.5 (13.9)	13.1 (15.2)
Smoking, *N* (%)		
Never	1013 (29.2)	616 (28.7)
Ex-smoker	1912 (55.1)	1188 (55.5)
Current smoker	543 (15.7)	335 (15.7)
Medication, *N* (%)		
Blood pressure-lowering medication	1534 (44.2)	838 (39.2)
Lipid-lowering medication	775 (22.3)	504 (23.7)
Cardiac medication	314 (9.1)	122 (5.7)
History of diseases, *N* (%)		
Diabetes mellitus	438 (12.6)	267 (12.5)
Left ventricular hypertrophy	192 (5.5)	118 (5.5)
Heart failure	129 (3.7)	37 (1.7)
Coronary heart disease	338 (9.7)	145 (6.8)
Hypertension	2775 (80.0)	1595 (74.6)
DXA measures		
Fat mass (kg)	26.41 (8.76)	–
Percentage of fat mass (%)	36.2 (9.3)	–
Lean mass (kg)	46.81 (9.25)	–
Percentage of lean mass (%)	63.9 (9.3)	–
Gynoid fat mass (kg)	4.05 (1.41)	–
Percentage of gynoid fat (%)	6.2 (2.2)	–
Android fat mass (kg)	2.51 (0.94)	–
Percentage of android fat (%)	3.4 (1.1)	–
Total body mass (kg)	73.22 (12.70)	–
Android to gynoid fat ratio	0.64 (0.20)	–
CT measures		
Liver attenuation (HU)	–	61.6 (55.1–65.6)
Epicardial fat volume (ml)	–	108.5 (40.3)

Values are showed as mean (standard deviation) or median (interquartile range) for continuous variables and number (percentage) for categorical variables

Abbreviations: *DXA*, Dual-energy X-ray absorptiometry; *CT*, computed tomography; *CVD*, cardiovascular disease; *BMI*, body mass index; *HDL*, high-density lipoprotein; *WHR*, waist-to-hip ratio

### Association of different fat depots with incident AF

Table 2 and Table 3 shows HRs with 95% CIs for associations between fat depots and incident AF. After adjustment for cardiovascular risk factors (model 2), higher levels of fat depots were significantly associated with new-onset AF with the exception for liver fat. The multivariable-adjusted HRs and 95% CIs were 1.22 (1.09–1.36) for fat mass, 1.14 (1.03–1.27) for android mass, 1.26 (1.13–1.41) for gynoid fat mass, and 1.27 (1.09–1.48) for epicardial fat. Notably, android-to-gynoid fat mass ratio showed an inverse association with incident AF [0.85 (0.74–0.98)]. After further adjustment for total lean mass (model 3), the associations between total fat mass [1.17 (1.04–1.32)], gynoid fat mass [1.21 (1.08–1.37)], and android-to-gynoid fat ratio [0.84 (0.72–0.97)] with incident AF remained statistically significant. In contrast, adjusting for total fat mass (model 4) substantially attenuated the significant associations between fat depots and AF, resulting in a borderline significant association between gynoid fat and AF only [1.36 (0.96–1.93)]. However, one needs to consider that model 4 could include highly significant correlations between the various fat depots and total fat mass and is therefore at the risk of overadjustment (Additional file 1: Fig. S1).

**Table 2:** Associations between various DXA-based fat depots and incident atrial fibrillation

DXA-based (***N*** = 3468)	Hazard ratio (95% confidence interval)			
	Model 1	Model 2	Model 3^a^	Model 4^b^
Total fat mass	1.28 (1.15–1.42)	1.22 (1.09–1.36)	1.17 (1.04–1.32)	–
Fat mass percentage	1.22 (1.07–1.41)	1.19 (1.03–1.37)	–	–
Android fat mass	1.20 (1.09-1.32)	1.14 (1.03–1.27)	1.06 (0.94–1.19)	0.78 (0.60–1.02)
Android fat percentage	1.09 (0.99–1.21)	1.06 (0.95–1.18)	1.04 (0.93–1.16)	–
Gynoid fat mass	1.31 (1.18–1.46)	1.26 (1.13–1.41)	1.21 (1.08–1.37)	1.36 (0.96–1.93)
Gynoid fat percentage	1.28 (1.09–1.49)	1.25 (1.07–1.47)	1.28 (1.09–1.50)	–
Android-to-gynoid fat ratio	0.93 (0.81–1.06)	0.85 (0.74–0.98)	0.84 (0.72–0.97)	0.82 (0.70–0.95)

Values are shown as hazard ratios and 95% confidence interval per 1 standard deviation increase of corresponding fat depots

*Abbreviations DXA* Dual-energy X-ray absorptiometry

Model 1 was adjusted for sex and age

Model 2 was additionally adjusted for high-density lipoprotein cholesterol, total cholesterol, smoking, total alcohol intake, lipid-lowering medication and cardiac medication and history of hypertension, left ventricular hypertrophy, diabetes mellitus, heart failure, and coronary heart disease

^a^Model 3: Model 2 + total lean mass

^b^Model 4: Model 2 + total fat mass

**Table 3:** Associations between various CT-based fat depots and incident atrial fibrillation

CT-based	Hazard ratio (95% confidence interval)			
	Model 1 (***N*** = 2139)	Model 2 (***N*** = 2139)	Model 3^a^ (***N*** = 1297)	Model 4^b^ (***N*** = 1297)
Liver fat	1.10 (0.94–1.28)	1.06 (0.90–1.25)	1.08 (0.88–1.32)	1.05 (0.86–1.29)
Epicardial fat	1.31 (1.13–1.51)	1.27 (1.09–1.48)	1.16 (0.95–1.42)	1.09 (0.87–1.38)

Values are shown as hazard ratios and 95% confidence interval per 1 standard deviation increase of corresponding fat depots

*Abbreviations CT* computed tomography

Model 1 was adjusted for sex and age

Model 2 was additionally adjusted for high-density lipoprotein cholesterol, total cholesterol, smoking, total alcohol intake, lipid-lowering medication and cardiac medication and history of hypertension, left ventricular hypertrophy, diabetes mellitus, heart failure, and coronary heart disease

^a^Model 3: Model 2 + total lean mass (in a sub-sample of 1297 participants with available DXA measurements)

^b^Model 4: Model 2 + total fat mass (in a sub-sample of 1297 participants with available DXA measurements)

In sensitivity analyses, no significant sex differences were observed for associations of all fat depots with incident AF (Additional file 1: Table. S1). In addition, higher values of epicardial fat conferred higher risks for AF among normal-weight participants, compared to overweight participants (*P* for interaction <0.01) (Additional file 1: Table. S2). After excluding participant with prevalent cardiovascular diseases (CHD, HF, and stroke) at baseline, we observed a slightly stronger association between each body fat depot and AF (Additional file 1: Table. S3). Finally, for the potentially intricated correlations between fat mass and lean mass, we also examined the association between total lean mass and incident AF (Additional file 1: Table. S4). In model 3 (fully adjusted model), higher lean mass was significantly associated with higher AF risk [1.31 (1.08-1.59)].

### Body fat score and incident AF

**Fig. 2:**
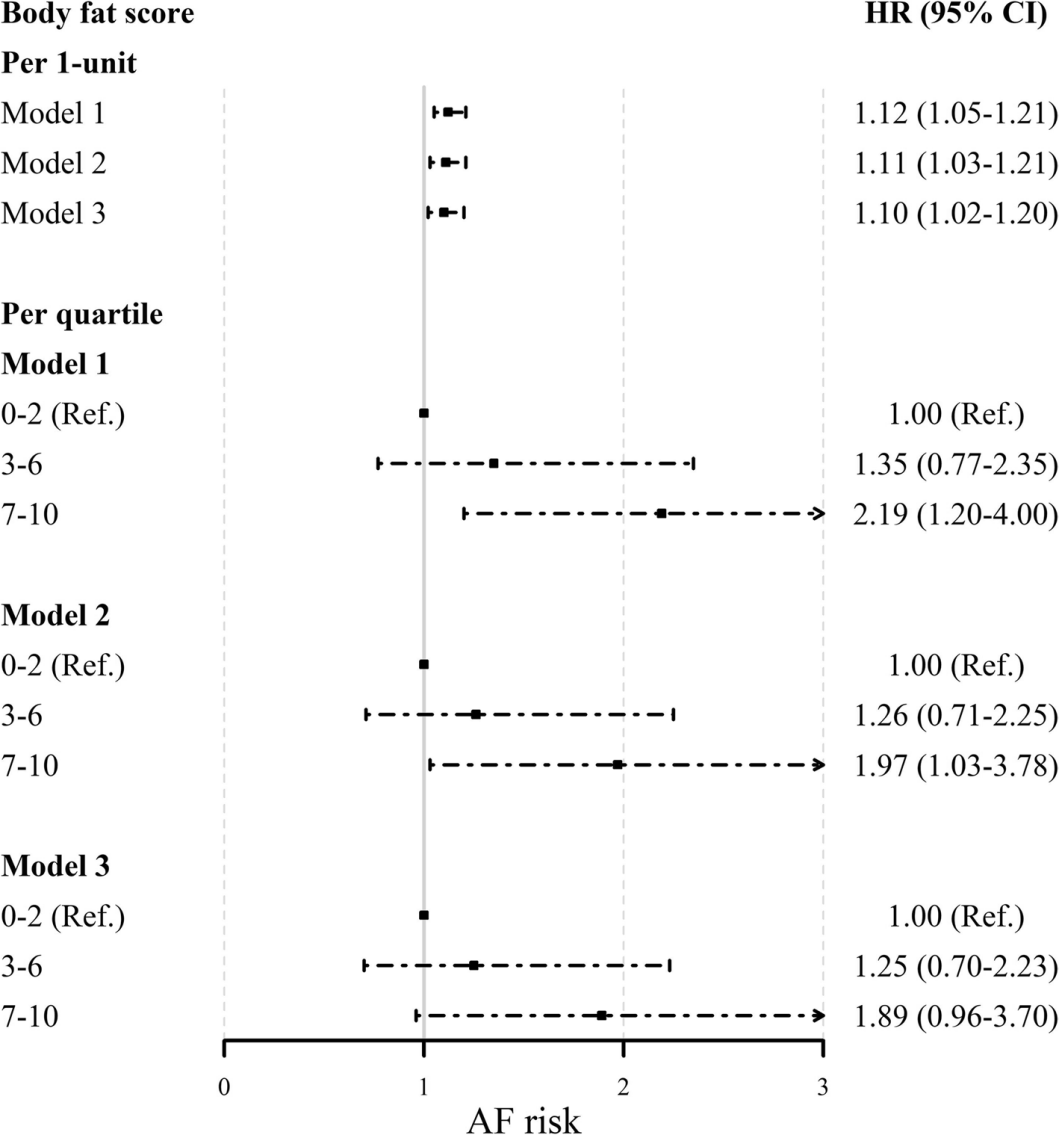
Association between body composition score and new-onset AF (*N* = 1297). Values are shown as hazard ratios (HR) and 95% confidence interval (95%CI). Model 1 was adjusted for age and sex. Model 2 was additionally adjusted for high-density lipoprotein (HDL) cholesterol, total cholesterol, smoking, total alcohol intake, systolic blood pressure, use of blood pressure-lowering medication, lipid-lowering medication and cardiac medication, and history of left ventricular hypertrophy, diabetes mellitus, heart failure, and coronary heart disease. Model 3 was additionally adjusted for total lean mass

Figure 2 depicts body fat score and its association with incident AF. Body fat score was significantly associated with incident AF after adjustments for age and sex (*P* for trend <0.001). Additional adjustment for traditional cardiovascular risk factors did not change the significance (*P* for trend <0.01). After further adjustment for total lean mass, higher body composition score was independently associated with higher risk of AF development [HR (95% CI) per 1-unit increase of fat score: 1.10 (1.02–1.20)].

### Fat distribution and incident AF

Based on PCA, two fat distribution patterns with an Eigen value of >1.0 were identified (scree plot in Additional file 1: Fig. S2) with a cumulative explained variance of 83%, namely: (1) a subcutaneous fat dominant pattern, characterized by high levels of total fat mass and gynoid fat mass; (2) a visceral fat dominant pattern, characterized by high levels of epicardial fat and liver fat. Factor loadings of each fat depot in two distribution patterns are shown in Additional file 1: Table. S5.

Table 4 shows HRs with 95%CI for the associations between two identified fat distribution patterns and new-onset AF. After adjustment for traditional cardiovascular risk factors (model 2), the subcutaneous fat dominant pattern was significantly associated with higher risk of incident AF, the HR (95% CI) per 1-SD increase of factor scores was 1.24 (1.02–1.51). However, after further adjustment for lean mass, the association attenuated to statistically null [HR (95% CI) 1.21 (0.98-1.49)]. In contrast, no association was found for the visceral fat dominant pattern with incident AF.

**Table 4:** Associations between fat depots patterns, identified through principal component analysis, with incident atrial fibrillation

	Per SD of factor scores	Per quartile of factor scores				*P* for trend
		Q1	Q2	Q3	Q4	
Subcutaneous fat dominant pattern (PC1)						
Model 1	1.27 (1.06–1.53)	1.00 (Ref.)	0.83 (0.47–1.45)	1.35 (0.81–2.25)	1.72 (1.00–2.94)	0.02
Model 2	1.24 (1.02–1.51)	1.00 (Ref.)	0.75 (0.43–1.35)	1.27 (0.75–2.13)	1.58 (0.91–2.74)	0.04
Model 3	1.21 (0.98–1.49)	1.00 (Ref.)	0.76 (0.43–1.34)	1.25 (0.74–2.11)	1.50 (0.85–2.65)	0.07
Visceral fat dominant pattern (PC2)						
Model 1	1.20 (0.99–1.50)	1.00 (Ref.)	1.77 (0.95–3.30)	2.07 (1.11–3.85)	1.86 (0.97–3.55)	0.09
Model 2	1.16 (0.93–1.47)	1.00 (Ref.)	1.83 (0.97–3.45)	1.90 (0.99–3.63)	1.69 (0.85–3.38)	0.20
Model 3	1.13 (0.90–1.43)	1.00 (Ref.)	1.82 (0.96–3.42)	1.84 (0.96–3.53)	1.58 (0.78–3.18)	0.31

Values are shown as hazard ratios and 95% confidence interval. *N* = 1297

*Abbreviations*: *SD* standard deviation, *PC* principal component

Model 1 was adjusted for age and sex. Model 2 was additionally adjusted for high-density lipoprotein cholesterol, total cholesterol, smoking, total alcohol intake, lipid-lowering medication and cardiac medication and history of hypertension, left ventricular hypertrophy, diabetes mellitus, heart failure, and coronary heart disease. Model 3 was additionally adjusted for total lean mass

## Discussion

In this prospective population-based study, we firstly assessed the associations between various body fat depots and new-onset AF after taking the total lean mass into account. Findings confirmed that various body fat depots were positively associated with new-onset AF after adjusting for traditional cardiovascular risk factors. Our study added new information by pointing out the associations between android fat, gynoid fat, and android-to-gynoid fat ratio with new-onset AF, even after taking body size into account. Although total fat mass is highly correlated with various fat depots, we further examined the additional values of individual fat depots on incident AF beyond total fat mass. Interestingly, android-to-gynoid fat mass ratio was inversely associated with incident AF regardless of total fat mass, implying a predominant role for subcutaneous fat on AF onset.

In line with the previous studies [8, 9, 33], we confirmed the positive associations between fat mass and fat mass percentage with incident AF. The causal roles of total fat mass have also been confirmed by a recent Mendelian randomization study [12]. Potential mechanisms for these associations include hypertension, volume overload, left ventricular diastolic abnormalities, autonomic dysfunction, and enhanced neurohormonal activation, which further contribute to increased atrial size and AF development [34, 35]. Moreover, excessive fat tissues are closely related to low-grade inflammation which is strongly associated with AF. Recent evidence suggests an important role for body size, rather than total fat mass, on AF development [8, 9, 36]. Typically, larger body size is closely associated with the increased left atrial size [37]. The latter is the most common cardiac structural remodelling in AF pathophysiology [38]. However, our findings presented robust associations between total fat mass and various fat depots with AF after adjusting for measures of body size, including total lean mass and left ventricular hypertrophy.

We, for the first time, showed an inverse association between android-to-gynoid fat ratio with incident AF. Also, our findings indicate that gynoid fat mass, but not android fat mass, is an independent risk factor for incident AF. Android fat is the adipose tissue mainly around the trunk including visceral fat. In contrast, gynoid fat is mainly the subcutaneous adipose tissue around the hip. Given participants in the current study tended to be older, the mechanisms to explain the association between android-gynoid ratio and AF might mostly relate to differential function of visceral and subcutaneous adipose tissue with aging. Adipose tissue undergoes critical changes to its inflammatory properties and cellular senescence as people grow older [20, 39]. Evidence suggests that inflammation in the elderly is more linked with subcutaneous adipose tissue than visceral adipose. Meanwhile, among subcutaneous adipose depots only, senescent cell burden may increase due to the shortened telomere length with aging [20, 39]. Herein, it seems that subcutaneous adipose tissue can have more detrimental impact on health than visceral fat tissue among the elderly. Therefore, gynoid fat might confer a higher risk of AF compared with android fat among the elderly; however, potential mechanisms warrant further investigations. In addition, our findings indicated that the body fat distribution pattern predominated by fat mass and gynoid fat mass had a stronger association with incident AF, compared to a weaker association for the pattern predominated by visceral fat mass. Taken together, our findings highlighted the potential benefit of assessing subcutaneous fat in the context of AF prevention, especially among the elderly.

Our findings suggested that both liver fat and epicardial fat are not independent risk factors for AF in the general population after adjustment for body size. Aforementioned, inflammation in the elderly is more linked with subcutaneous adipose tissue than visceral adipose which also supports our findings that visceral adipose conferred limited impact on AF development. Consistently, the Framingham Heart Study similarly indicated that there was no significant impact of visceral fat tissue and hepatic steatosis on incident AF [14, 15]. Within the Rotterdam Study cohort, previous research has shown a significant association between epicardial fat and AF among participants free of CVD [13]. This implies that prevalent CVD may, to some extent, impede the effect of epicardial fat on AF. In our sensitivity analyses among population free of prevalent heart failure, coronary heart disease and stroke, we indeed found stronger associations between various fat depots and incident AF.

### Study strengths and limitations

Strengths of our study include its prospective design, the long follow-up, and adequate adjustment for a broad range of confounders. AF events were meticulously adjudicated, validated, and confirmed on electrocardiogram. Furthermore, availability of multiple DXA- and CT-assessed fat depots allowed for comprehensive investigation and comparison of the association of various body fat depots with new-onset AF. Our study adds to the previous research by demonstrating a positive association between gynoid fat and incident AF, as well as an inverse association between android-to-gynoid fat ratio and incident AF. Of note, findings indicated a robust and independent association between gynoid fat and android-to-gynoid fat ratio with AF after adjusting for BMI, WHR, or total lean mass, (Additional file 1: Table. S6) reflecting additional predictive information regarding assessing body fat depots for AF beyond traditional anthropometric markers and measures of body size. However, several limitations of this study need to be acknowledged. First, the majority of our participants were of European ancestry and older adults, limiting the generalizability of our findings to younger populations and other ethnicities. Second, though we adjusted for many potential confounders, given the observational study design, we cannot rule out the possibility of residual or unmeasured confounding. Third, our study was based on single (baseline) imaging measures to assess body fat. Thus, the potential impact of changes of body fat over time on AF development could not be taken into account. Finally, since AF may be paroxysmal and asymptomatic, we might have underestimated the true number of AF cases in our study population. However, it is estimated that more than 75% of AF cases among the European population are permanent or persistent AF and most paroxysmal AF cases end as the permanent form [40]. In addition, the prevalence of AF in the Rotterdam Study is ~4% which is in line with the global estimate of AF prevalence [2].

## Conclusions

Various body fat depots were associated with new-onset AF. Gynoid fat mass was independently associated with AF after taking body size into account. The inverse association between android-to-gynoid fat ratio with new-onset AF presents a novel finding. A significant dose-response relationship between body fat accumulation and incident AF was observed. Our results underscored a predominant role for subcutaneous fat in AF development.

## Supplementary Information

**Additional file 1: Methods.** Assessment of cardiovascular risk factors. **Table S1.** Associations between various fat depots and incident atrial fibrillation among men and women. **Table S2.** Associations between various fat depots and incident atrial fibrillation, stratified by body mass index. **Table S3.** Associations between various fat depots and incident atrial fibrillation among participants free of prevalent cardiovascular disease . **Table S4.** Associations between lean body mass and incident atrial fibrillation. **Table S5.** Factor loadings for each fat distribution pattern in principal component analysis . **Table S6.** Associations between various fat depots and incident atrial fibrillation among men and women adjusting for body mass index and waist-to-hip ratio. **Figure S1.** Correlations between various fat depots. **Figure S2.** Scree plot in principal component analysis.

## Data Availability

Data can be obtained upon request. Requests should be directed towards the management team of the Rotterdam Study (secretariat.epi@erasmusmc.nl), which has a protocol for approving data requests. Because of restrictions based on privacy regulations and informed consent of the participants, data cannot be made freely available in a public repository.

## Abbreviations

AF: Atrial fibrillation
BMI: Body mass index
CI: Confidence intervals
CT: Computed tomography
DXA: Dual-energy X-ray absorptiometry
ECG: Electrocardiogram
HR: Hazard ratio
HU: Hounsfield unit
LVH: Left ventricular hypertrophy
PCA: Principal component analysis
RS: Rotterdam Study
SBP: Systolic blood pressure
SD: Standard deviation
