# Supplementary material for: Imaging-based body fat depots and new-onset atrial fibrillation in general population: a prospective cohort study
Source: BMC Med. 2022 Sep 19;20:317. doi: 10.1186/s12916-022-02505-y (PMC9484252; doi:10.1186/s12916-022-02505-y)
Supplement: Supplementary file 1 — Additional file 1: Methods. Assessment of cardiovascular risk factors. Table S1. Associations between various fat depots and incident atrial fibrillation among men and women. Table S2. Associations between various fat depots and incident atrial fibrillation, stratified by body mass index. Table S3. Associations between various fat depots and incident atrial fibrillation among participants free of prevalent cardiovascular disease . Table S4. Associations between lean body mass and incident atrial fibrillation. Table S5. Factor loadings for each fat distribution pattern in principal component analysis . Table S6. Associations between various fat depots and incident atrial fibrillation among men and women adjusting for body mass index and waist-to-hip ratio. Figure S1. Correlations between various fat depots. Figure S2. Scree plot in principal component analysis. [file 12916_2022_2505_MOESM1_ESM.docx]

**Additional file for the manuscript:**

Title: Imaging-based body fat depots and new-onset atrial fibrillation in the general population

Brief title: Body fat depots and incident atrial fibrillation

Authors: Zuolin Lu^a^, Martijn J. Tilly^a^, Elif Aribasa, Daniel Bos^a,b^, Sven Geurts^a^, Bruno H. Stricker^a^, Robert de Knegt^a,c^, M. Arfan Ikram^a^, Natasja de Groot^d^, Trudy Voortman^a^, Maryam Kavousi^a^

Affiliations:

a Department of Epidemiology, Erasmus MC, University Medical Centre Rotterdam, Rotterdam, The Netherlands

b Department of Radiology and Nuclear Medicine, Erasmus MC, Erasmus University Medical Centre Rotterdam, Rotterdam, The Netherlands

c Department of Gastroenterology & Hepatology, Erasmus MC, Erasmus University Medical Centre Rotterdam, Rotterdam, The Netherlands

d Department of Cardiology, Erasmus MC, Erasmus University Medical Centre Rotterdam, Rotterdam, The Netherlands

**Methods**

All participants responded to comprehensive computerized questionnaires at study baseline about their current health status, medical history, medication, and lifestyle. They were interviewed at home by trained interviewers and underwent more extensive clinical examination and laboratory assessments at the research center.

Serum total and high-density lipoprotein (HDL) cholesterol were measured with an automated enzymatic method. Blood pressure was measured twice at the right upper arm with a random zero mercury sphygmomanometer in the sitting position. Systolic and diastolic blood pressures were calculated as the mean of the two consecutive measurements. Hypertension was defined as a systolic blood pressure (SBP) of ≥140 mmHg or a diastolic blood pressure (DBP) ≥ 90 mmHg or use of blood pressure lowering drugs prescribed for hypertension [ATC-codes C02, C03, C07, C08, and C09].(20) Diabetes mellitus (DM) was defined as fasting serum glucose levels ≥ 126 mg/dL (7.0 mmol/L) (or non-fasting serum glucose levels ≥ 200 mg/dL (11.1 mmol/L) if fasting samples were unavailable) or the use of antidiabetic therapy [ATC-code A10]. Smoking information derived from baseline questionnaires was categorized into current, former and never smokers. Data on alcohol consumption were collected as part of a dietary interview and expressed in ethanol intake per day in grams. Left ventricular hypertrophy (LVH) was diagnosed using the MEANS program with an algorithm that takes into accounts QRS voltages, with an age-dependent correction and repolarization. The assessment and definition of prevalent coronary heart disease (CHD), stroke and heart failure (HF) has been described in detail previously. (25, 27) Information on lipid-lowering [ATC-code C10] and cardiac medication use was derived from baseline questionnaires and pharmacy data. Cardiac medication was defined as use of digoxin [ATC-code C01AA05], nitrates [ATC-code C01DA] or antiarrhythmic drugs [ATC-code C01B].


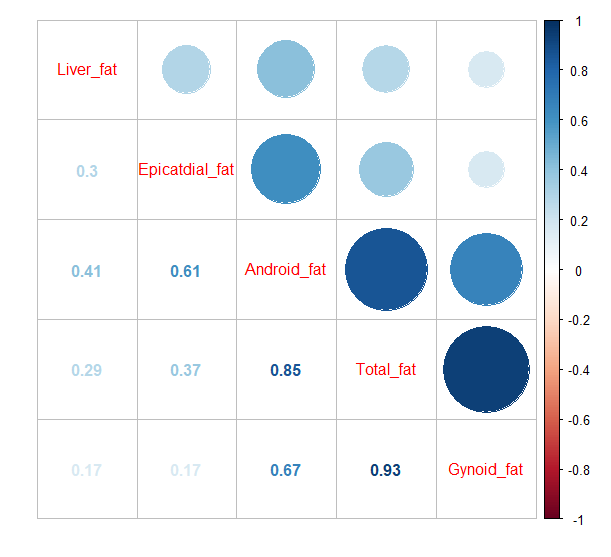
Figure S1. Correlations between various fat depots

| Table S1. Associations between various fat depots and incident atrial fibrillation among men and women | | | |
| --- | --- | --- | --- |
|  | Participants | | *P* for interaction |
|  | Men | Women |  |
| **DXA (n = 3468)** |  |  |  |
| Total fat mass | 1.25 (1.05-1.49) | 1.18 (1.02-1.37) | 0.48 |
| Fat mass percentage | 1.19 (0.96-1.47) | 1.15 (0.94-1.40) | 0.98 |
| Android fat mass | 1.18 (1.01-1.38) | 1.09 (0.94-1.27) | 0.65 |
| Android fat percentage | 1.07 (0.91-1.25) | 0.98 (0.84-1.14) | 0.51 |
| Gynoid fat mass | 1.31 (1.08-1.59) | 1.21 (1.05-1.40) | 0.56 |
| Gynoid fat percentage | 1.27 (0.98-1.64) | 1.16 (0.94-1.42) | 0.59 |
| Android-to-gynoid  fat ratio | 0.92 (0.77-1.10) | 0.82 (0.63-1.02) | 0.45 |
| **CT (n = 2139)** |  |  |  |
| Liver fat | 1.09 (0.87-1.36) | 1.02 (0.80-1.31) | 0.95 |
| Epicardial fat | 1.26 (1.04-1.52) | 1.28 (0.91-1.78) | 0.74 |
| Values are shown as hazard ratios (HR) and 95% confidence interval (95%CI).  Model was adjusted for age, high-density lipoprotein cholesterol, total cholesterol, smoking, total alcohol intake, lipid lowering medication and cardiac medication and history of hypertension, left ventricular hypertrophy, diabetes mellitus, heart failure and coronary heart disease. | | | |

| Table S2. Associations between various fat depots and incident atrial fibrillation, stratified by body mass index | | | |
| --- | --- | --- | --- |
|  | BMI categories | | *P* for interaction |
|  | BMI < 25 | BMI >= 25 |  |
| **DXA (N = 3468)** |  |  |  |
| Fat mass |  |  |  |
| Model 1 | 1.24 (0.80-1.91) | 1.18 (1.02-1.36) | 0.49 |
| Model 2 | - | - | - |
| Android fat mass |  |  |  |
| Model 1 | 1.19 (0.81-1.76) | 1.07 (0.93-1.22) | 0.69 |
| Model 2 | 1.02 (0.47-2.22) | 0.78 (0.59-1.04) | 0.99 |
| Gynoid fat mass |  |  |  |
| Model 1 | 1.18 (0.75-1.84) | 1.22 (1.07-1.41) | 0.28 |
| Model 2 | 0.87 (0.32-2.32) | 1.43 (0.98-2.09) | 0.23 |
| **CT (N = 2139)** |  |  |  |
| Liver fat |  |  |  |
| Model 1 | 1.54 (0.89-2.67) | 1.01 (0.81-1.25) | 0.22 |
| Model 2 | 1.46 (0.85-2.50) | 0.99 (0.79-1.24) | 0.23 |
| Epicardial fat |  |  |  |
| Model 1 | 2.36 (1.24-4.48) | 1.10 (0.87-1.39) | <0.01 |
| Model 2 | 1.93 (0.94-3.95) | 1.07 (0.82-1.39) | <0.01 |
| Values are shown as hazard ratios (HR) and 95% confidence interval (95%CI) per 1 standard deviation (SD) increase. Abbreviation: BMI, body mass index. Abbreviations: DXA, X-ray absorptiometry; CT, computed tomography.  Model 1 was adjusted for age, sex, high-density lipoprotein cholesterol, total cholesterol, smoking, total alcohol intake, lipid lowering medication and cardiac medication, history of hypertension, left ventricular hypertrophy, diabetes mellitus, heart failure and coronary heart disease, and total lean mass.  Model 2 was adjusted for age, sex, high-density lipoprotein cholesterol, total cholesterol, smoking, total alcohol intake, lipid lowering medication and cardiac medication, history of hypertension, left ventricular hypertrophy, diabetes mellitus, heart failure and coronary heart disease, and total fat mass. | | | |

| Table S3. Associations between various fat depots and incident atrial fibrillation among participants free of prevalent cardiovascular disease | | | | |
| --- | --- | --- | --- | --- |
|  | Hazard ratio (95% confidence intervals) | | | |
|  | Model 1 | Model 2 | Model 3* | Model 4† |
| **DXA study**  **(N = 3216)** |  |  |  |  |
| Fat mass | 1.28 (1.14-1.45) | 1.23 (1.08-1.40) | 1.17 (1.02-1.35) | - |
| Android fat mass | 1.19 (1.06-1.34) | 1.14 (1.01-1.29) | 1.07 (0.94-1.23) | 0.73 (0.54-1.00) |
| Gynoid fat mass | 1.32 (1.16-1.50) | 1.27 (1.11-1.45) | 1.22 (1.06-1.40) | 1.37 (0.91-2.07) |
| Android-to-gynoid  fat ratio | 0.91 (0.78-1.06) | 0.84 (0.71-0.99) | 0.82 (0.69-0.97) | 0.79 (0.66-0.94) |
|  |  |  |  |  |
| **CT study**  **(N = 1990)** |  |  |  |  |
| Liver fat | 1.19 (1.00-1.42) | 1.14 (0.95-1.37) | 1.12 (0.89-1.41)‡ | 1.06 (0.74-1.35)‡ |
| Epicardial fat | 1.36 (1.17-1.59) | 1.35 (1.14-1.59) | 1.27 (1.02-1.59)‡ | 1.20 (0.92-1.55)‡ |
| Values are shown as hazard ratios (HR) and 95% confidence interval (95% CI) per standard deviation (SD) increase. Abbreviations: DXA, X-ray absorptiometry; CT, computed tomography.  Prevalent cardiovascular disease was defined as prevalent coronary heart disease, heart failure, stroke, or AF at baseline.  Model 1 was adjusted for sex and age.  Model 2 was additionally adjusted for high-density lipoprotein cholesterol, total cholesterol, smoking, total alcohol intake, lipid lowering medication and cardiac medication and history of hypertension, left ventricular hypertrophy and diabetes mellitus.  *Model 3: Model 2 + total lean mass.  †Model 4: Model 2 + total fat mass.  ‡In a sub-sample of 1123 participants with available DXA measurements. | | | | |

| Table S4. Associations between lean body mass and incident atrial fibrillation | | | |
| --- | --- | --- | --- |
|  | Hazard ratio (95% confidence intervals) | | |
| **DXA study**  **(N = 3216)** | Model 1 | Model 2 | Model 3 |
| Total lean mass | 1.47 (1.24-1.75) | 1.45 (1.21-1.74) | 1.31 (1.08-1.59) |
| Lean mass percentage | 0.81 (0.71-0.93) | 0.84 (0.73-0.98) | - |
| Values are shown as hazard ratios (HR) and 95% confidence interval (95% CI) per standard deviation (SD) increase. Abbreviations: DXA, X-ray absorptiometry.  Model 1 was adjusted for sex and age.  Model 2 was additionally adjusted for high-density lipoprotein cholesterol, total cholesterol, smoking, total alcohol intake, lipid lowering medication and cardiac medication and history of hypertension, left ventricular hypertrophy and diabetes mellitus.  Model 3: Model 2 + total fat mass. | | | |

| Table S5. Factor loadings for each fat distribution pattern in principal component analysis | | |
| --- | --- | --- |
|  | Factor loadings (N = 1297) | |
|  | Principal component 1  (Subcutaneous fat pattern) | Principal component 2  (Visceral fat pattern) |
| Liver fat | 0.07 | **0.79** |
| Epicardial fat | 0.21 | **0.80** |
| Android fat | **0.74** | **0.58** |
| Gynoid fat | **0.97** | 0.02 |
| Fat mass | **0.96** | 0.25 |

Two patterns cumulatively explained 83% total variation.


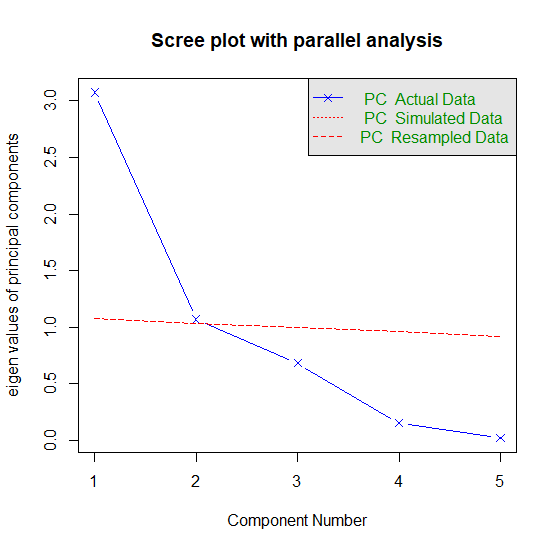


Figure S2. Scree plot in principal component analysis

| Table S6. Associations between various fat depots and incident atrial fibrillation, adjusting for body mass index and waist-to-hip ratio | | | |
| --- | --- | --- | --- |
|  | AF risk | | |
|  | Model 1 | Model 2 | Model 3 |
| **DXA (n = 3468)** |  |  |  |
| Total fat mass | 1.22 (1.09-1.36) | 1.33 (1.05-1.68) | 1.27 (1.13-1.42) |
| Fat mass percentage | 1.19 (1.03-1.37) | 1.19 (1.03-1.37) | 1.18 (1.01-1.37) |
| Android fat mass | 1.14 (1.03-1.27) | 1.05 (0.87-1.27) | 1.16 (1.03-1.32) |
| Android fat percentage | 1.06 (0.95-1.18) | 1.06 (0.95-1.18) | 1.03 (0.90-1.17) |
| Gynoid fat mass | 1.26 (1.13-1.41) | 1.36 (1.12-1.65) | 1.29 (1.15-1.44) |
| Gynoid fat percentage | 1.25 (1.07-1.47) | 1.25 (1.07-1.47) | 1.25 (1.07-1.47) |
| Android-to-gynoid fat ratio | 0.85 (0.74-0.98) | 0.81 (0.70-0.94) | 0.77 (0.65-0.91) |
| **CT (n = 2139)** |  |  |  |
| Liver fat | 1.06 (0.90-1.25) | 1.00 (0.85-1.19) | 1.07 (0.90-1.27) |
| Epicardial fat | 1.27 (1.09-1.48) | 1.18 (0.99-1.41) | 1.32 (1.11-1.57) |
| Values are shown as hazard ratios (HR) and 95% confidence interval (95% CI).  Model 1 was adjusted for age, high-density lipoprotein cholesterol, total cholesterol, smoking, total alcohol intake, lipid lowering medication and cardiac medication, history of hypertension, left ventricular hypertrophy, diabetes mellitus, heart failure and coronary heart disease.  Model 2: Model 1 + body mass index.  Model 3: Model 1 + waist-to-hip ratio. | | | |
